# Development and validation of Baritrip: a multidisciplinary educational mobile application for bariatric surgery patients

**DOI:** 10.31744/einstein_journal/2025AO1362

**Published:** 2025-08-25

**Authors:** Bruno Côrtes Gonçalves, Janice Sepúlveda Reis, Aleida Nazareth Soares, Débora Cardoso Rossi, Olivia Silva Eler, Alexandre Sampaio Moura

**Affiliations:** 1 Faculdade de Saúde Santa Casa de Belo Horizonte Belo Horizonte MG Brazil Faculdade de Saúde Santa Casa de Belo Horizonte, Belo Horizonte, MG, Brazil.

**Keywords:** Bariatric surgery, Mobile applications, Information technology, Health literacy, Public health Informatics, Health promotion

## Abstract

**Objective:**

To develop and validate a comprehensive mobile health App (mHealth App) to increase the health literacy and care engagement of patients undergoing bariatric surgery.

**Methods:**

App development required identifying patient needs, conducting a literature review, benchmarking, and designing the App through a multi-professional team. A prototype was created and validated by healthcare and information technology specialists, and patients undergoing bariatric surgery.

**Results:**

The Baritrip App contained 188 screens divided into eight sections, covering clinical, surgical, nutritional, mental health, and speech therapy domains. The overall content validity index of the App as assessed by experts was 0.99. Bariatric surgery patients assessed the layout, language, content organization, and engagement of the App, with an overall agreement rate of 89.1%.

**Conclusion:**

The Baritrip mobile health App showed good content validity and cultural appropriateness for bariatric surgery patients.

## In Brief

The Baritrip mHealth app was developed to improve health literacy, pre and post-surgical care of bariatric patients. A multiprofessional team designed and validated the app, ensuring comprehensive content covering nutrition, mental health, and speech therapy domains. Expert validation showed high reliability (CVI: 0.99), and patients rated its usability and engagement positively (89.1% agreement).

## Highlights

■ The Baritrip app offers multidisciplinary guidance for bariatric surgery patients.
■ The app was validated by experts, yielding a high content validity index of 0.99.
■ Users rated the app engagement, layout, and organization with 89.1% agreement.
■ The app supports health literacy, pre and post-surgical care through an inclusive approach.

## Graphical abstract

**Figure:**
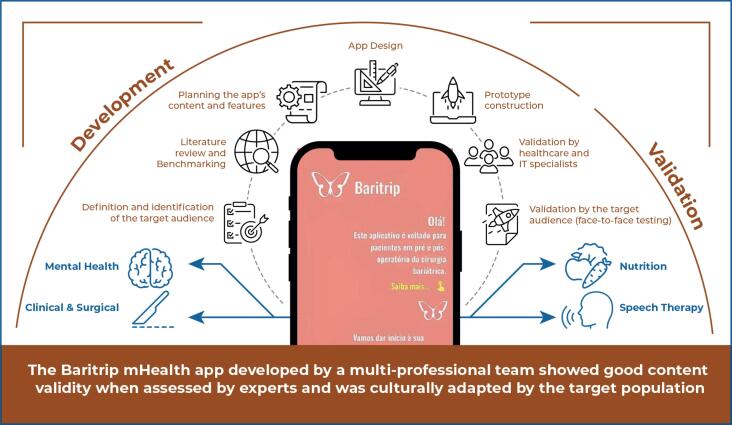

## INTRODUCTION

Obesity is recognized as a global epidemic and one of the most serious public health problems, affecting approximately 30.0% of the global population.^(1–3)^ Over the last 40 years, the prevalence of overweight and obesity has quadrupled, increasing the risk for diabetes, cardiovascular disease, and certain cancers.^(4)^

Bariatric surgery has emerged as the most effective treatment for severe obesity,^(5)^ providing a significant reduction in obesity-related complications such as type 2 diabetes and cardiovascular disease.^(1)^ Despite its popularity and effectiveness, weight loss after bariatric surgery varies, with approximately 30.0% of patients experiencing weight regain after surgery.^(6)^ Contributing factors include poor eating habits, reduced physical activity, and hormonal adaptations.^(7)^ Patients undergoing bariatric surgery face daily challenges of learning how to adapt to their new lifestyle and restore their health.^(8,9)^ Providing information and intensive follow-ups are important to enable them to engage in effective self-care.^(10)^

Health literacy refers to the ability of a patient to comprehend the necessary health information to make informed decisions.^(11)^ Improving health literacy among patients undergoing bariatric surgery is essential because this can influence adherence to healthcare appointments and engagement in followups.^(12)^ To promote health equity, strategies to enhance health literacy must be contextually adapted, and innovative technologies, such as mobile health (mHealth) Apps, offer promising solutions.^(13)^

In Brazil, healthcare providers within the Brazilian Unified Health System (SUS - *Sistema* Único *de Saúde*) face challenges due to inadequate patient health literacy.^(14)^ There is a noticeable gap in the medical literature addressing strategies to enhance health literacy among SUS users.

The popularization of mHealth Apps has brought new perspectives to patient education and care engagement. Patients undergoing bariatric surgery have shown an interest in using mHealth Apps that promote education and engagement in care. Studies have shown that patients using such Apps may experience greater weight loss and reduced weight gain.^(15)^ Hence, developing an educational instrument as an mHealth App to deliver accurate patient-centered information is a promising strategy.

Despite this potential, mHealth Apps targeting patients undergoing bariatric surgery are scarce in Portugal. Among the few available, Barilife is the most popular. It provides a platform wherein bariatric surgery patients can identify patterns and trends that enhance their overall user experience. Barilife provides instructional videos from healthcare professionals and offers a range of tools and resources designed for patients undergoing bariatric surgery; however, it was not tailored for SUS patients and lacks appropriate validation.

## OBJECTIVE

This study aimed to develop and validate an mHealth App to increase the health literacy and self-care of patients undergoing bariatric surgery in the Brazilian Unified Health System, incorporating input from multidisciplinary experts and the target population.

## METHODS

### Study design

**Figure 1:**
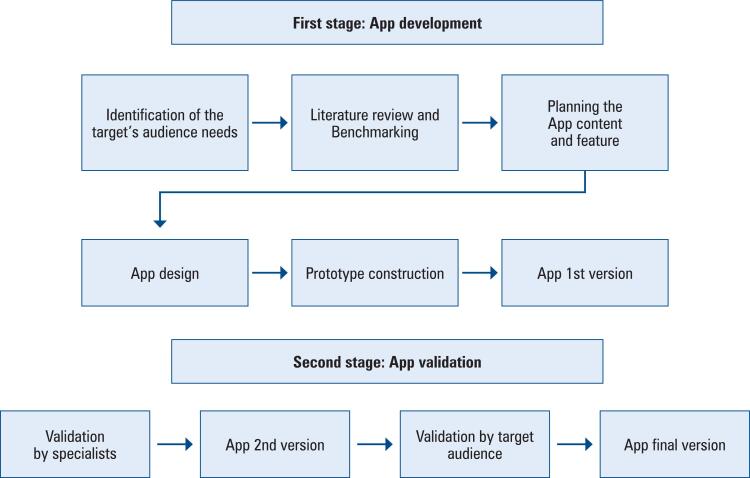
App's development and validation flowchart

The study was conducted in two stages comprising of multiple steps, as outlined in figure 1.

The first stage was App development, while the second stage was content validation by healthcare and information technology (IT) experts, and the target population. This study was approved by the Ethics Committee of *Santa Casa de Belo Horizonte* (CAAE: 87762218.6.0000.5138; #6.179.496), and all participants provided written informed consent.

This study was conducted at the Medical Specialties Center of *Santa Casa Belo Horizonte* (CEM-SCBH), Brazil, a public multiprofessional outpatient care facility for patients with obesity.

## PROCEDURE

### First stage – App development 1^st^ Step – Definition and identification of the target audience

The App was designed for patients undergoing preoperative and postoperative care for bariatric surgery in the Brazilian Unified Health System. The target audience was identified based on behavioral, cultural, social, and financial characteristics.

Preoperative participants included those who attended at least one appointment at the CEMSCBH and had a body mass index (BMI) ≥40 without comorbidities or ≥35 with comorbidities.^(16)^ Postoperative participants included those who underwent bariatric surgery at CEM-SCBH, regardless of BMI or time since surgery. All participants were aged 18–65 years.

A three-item questionnaire was administered to 15 patients who received preoperative and postoperative care at CEM-SCBH: (1) "If you were to create an App for bariatric patients, which content and features would you like to include?" (2) "What tasks do you hope the App will help you with?" (3) "Do you have any additional suggestions or ideas for the App?" This step helped identify content that users considered important for the App.

### 2^nd^ Step – Literature review and benchmarking

To define the ideas and goals of the application, an integrative literature review focusing on preoperative and postoperative care in bariatric surgery was conducted, including 88 publications, to supplement the data obtained in the initial step and define the scope of the App.

A benchmarking process was used to identify competitors and evaluate existing Apps in the Google Play, Apple Store, and PubMed between July 2021 and September 2021. The following keywords were used: "bariatric surgery," "weight loss surgery," "gastric band," "gastric sleeve," "gastric bypass," "obesity," "mobile application," and "App." This review targeted applications that monitor hydration levels throughout the bariatric surgery journey, facilitate physical activity routines, provide diet alerts, and assist in appointment scheduling.

### 3^rd^ Step – Planning App content and features

A multi-professional expert committee was created to develop the App content. The committee comprised of ten professionals from CEM-SCBH, including two endocrinologists, one bariatric surgeon, one plastic surgeon, one nutritionist, two psychologists, one nurse, and two speech therapists. Each professional had more than 5 years of experience in preoperative and postoperative bariatric care.

Content development followed the recommendations for preparing healthcare manuals.^(17)^ The committee met three times to discuss the content and features of the App, ensuring alignment with user needs identified during the audience assessment.

All technical content was documented using Microsoft PowerPoint to articulate and visually represent key concepts and workflows. This documentation was continuously updated throughout development, undergoing regular reviews and updates to incorporate new insights and adjustments. Upon completion of the third step, the committee convened to review the content and agreed to advance to the fourth step.

### 4^th^ Step – App design

The layout and visual elements of the App were designed based on the characteristics of the target audience and expert committee decisions to increase the clarity and applicability of the content. The App design included color schemes, labels, and transitions using the Figma^®^ platform, an intuitive, real-time collaboration tool that enables an efficient and dynamic workflow to test App designs.

### 5^th^ Step – Prototype construction

App prototyping was conducted in partnership with an IT professional who was experienced in the development of web and mobile applications. A high-fidelity, functional prototype was built using the Adalo^®^ platform, which is designed to create personalized applications without coding and produce applications that are compatible to different devices such as desktops and mobile devices. Adalo^®^ has a rapid development framework that allows flexible construction of screens using widgets.^(18,19)^

An application prototype was created during the development stage with a home screen titled "Your Journey," with icons for accessing preoperative and different stages of postoperative care.

Beginning with the initial presentation screen, the App featured eight sections. The starting section presented a checklist of consultations and examinations necessary to prepare for the preoperative phase. The next section presented the preoperative content with information about the surgery and guidance on preoperative medical, nutritional, and mental health care. The three following sections were related to postoperative care divided into three different periods (0–12, 12–24, and ≥24 months). Each postoperative period presented medical, nutritional, mental health, and speech therapy information, as well as a consultation schedule.

The "For your entire journey" section presented practical recommendations for all postoperative periods, including sleep hygiene, protein intake, and relaxation techniques. The last section ("Supplementary material") emphasizes long-term lifestyle changes and provides strategies to prevent weight regain and control anxiety.

The follow-up period was defined such that patients were monitored for at least 24 months post-surgery. It is crucial to note that inadequate weight loss tends to increase in the years following surgery, underscoring the need for extended monitoring.^(20)^

A vital function of the application is to promote self-care in patients undergoing bariatric surgery to mitigate weight regain. The application covers the following topics: healthy diet, physical exercise, medication adherence, complication management and prevention, stress management, medical appointments, and sleep hygiene. These features were designed to enhance patient engagement.

The expert committee held one meeting to review and approve the prototype for validation. After completing the five steps in the first stage, the first version of the application was established.

### Second stage – App validation

The second stage involved validation by judges, the expert committee, and target audience. The selection of the judges followed established guidelines, ensuring participation from at least three healthcare specialists and three IT experts^(21–23)^ who met at least two criteria of expertise.^(24)^

The panel of judges included seven healthcare specialists experienced in bariatric surgery care and in health education, and five IT experts experienced in developing mobile applications and software. Healthcare specialists from the CEM-SCBH were not included as judges.

### 1^st^ Step – Validation by healthcare and IT specialists

The first step in the validation stage was conducted using an online Google Forms questionnaire. The judges were contacted via email and invited to evaluate each section of the App using heuristics as proposed by D’ Carlo et al.^(25)^ Judges received instructions on how to use the App and answer the questionnaire.

The Content Validity Index (CVI) was calculated to evaluate the inter-rater agreement. The CVI was determined by the proportion of responses rated as "3: optional modification" and "4: does not need modification" relative to the total responses. Items rated as "1: need for complete modification" and "2: need for partial modification" were flagged for revision or elimination.

The overall CVI exceeded the defined cutoff point of 0.78; hence, the committee reviewed and discussed the qualitative feedback from the judges. The committee justified changes that were accepted and rejected. After modifications, the second prototype was produced. Validation and modifications took 6 months to complete.

### 2^nd^ Step – Validation by the target audience (face-to-face testing)

Preoperative and postoperative bariatric surgery patients from CEM-SCBH were selected for face-to-face testing. All selected patients were followed up at CEM-SCBH. Validation and cultural adaptation involved 23 patients.

The target audience validated the content of the prototype and assessed its clarity and relevance. Comments and suggestions were provided whenever necessary. Testing was conducted face-to-face in group sessions with at least five patients in each round. Items were reviewed if 15% or more participants found them difficult to understand.^(26)^ New patient groups were selected for each round, and the App was adjusted between rounds. After three rounds, no further changes were suggested, resulting in the final App prototype.

## RESULTS

The first stage of the study was conducted between October 2021 and January 2023. In the first step, 15 preoperative and postoperative patients were interviewed regarding their needs. Of these, 12 (80.0%) were female and 9 (60.0 %) were in the preoperative stage. All 15 patients (100%) had smartphones, and 11 (73.0%) had previously used a weight tracker application. A summary of the patient needs is presented in table 1.

**Table 1:** User suggestions for App content

Topic	Summary of suggestions
Surgery characteristics	Information about the different types of surgery; Criteria for undergoing surgery; Information on how much weight loss is expected after bariatric surgery; Information on who can undergo and timing of post-bariatric plastic surgery
Clinical issues	Strategies to prevent weight regain; Possible surgical complications; Strategies to prevent hair loss after bariatric surgery; Information on the symptoms of Dumping Syndrome
Mental health issues	Information on how to deal with preoperative and postoperative anxiety
Nutritional issues	Information on the adequate protein intake for postoperative patients; Information on postoperative diet and vitamin replacement
Reminders	Reminder to drink water; Reminder of appointment schedule; Reminders on preoperative and postoperative exams

After assessing the needs of patients, a literature review was performed, and 88 relevant manuscripts were selected. The review demonstrated a lack of multidisciplinary approaches for bariatric surgery care and promoting health literacy. Thirteen Apps were analyzed for benchmarking, which showed the potential of some Apps to improve accessibility, knowledge, and self-care among bariatric surgery patients.

The Mobile App Classification Benchmark Summary included several App categories. Bariatric surgery and post-operative care Apps included BariatricPal, Baritastic App, Barilife, BariUFU, BariBuddy, and Bariatric IQ.^(27)^ Nutrition and diet management apps included Foodeducate, Plate, and PromMera.^(28)^ Fitness and health tracking Apps included Waterlogged, My Diet Coach, Happiness Scale, Fitbit & Aria, and PromMera. A comparative benchmark of these Apps is presented in table 2.

**Table 2:** Comparative benchmarking of mobile health applications for bariatric surgery patients

App	Language	Validation	Trial	Physical activity	Nutrition	Speech therapy	Psychologist
BariUFU	Portuguese				√		√
Barilife	Portuguese			√	√		
PromMera	English		√	√	√		
Baritastic	English			√	√		
BariatricPal	English				√		
BariBuddy	English			√	√		
Bariatric IQ	English				√		
Foodeducate	English				√		
Plate	English				√		
Waterlogged	English/ Portuguese				√		
My Diet Coach	French				√		
Happy Scale	English				√		
Fitbit & Aria	Multiple languages				√		

Subsequently, content and features for the App were developed in collaboration with an expert committee of 10 professionals with diverse expertise relevant to the study objectives, 80.0% of whom were female. Among them, 20% had a master's or doctoral degree, 70.0% had specialized training, and 50.0% had over 14 years of experience. The team included four physicians (40.0%), two psychologists (20.0%), two speech therapists (20.0%), one nutritionist (10.0%), and one nurse (10.0%), ensuring comprehensive and well-rounded input for App development.

The content and features of the App were evaluated by a panel of 12 specialists. Among them, five (41.7%) were female, eight (66.7%) had a master's or doctoral degree, and eight (66.6%) had over 14 years of experience. The team included five IT professionals (41.7%), three physicians (25.0%), one nutritionist (8.3%), one psychologist (8.3%), one speech therapist (8.3%), and one nurse (8.3%). Of the IT professionals, three (60.0%) were academic researchers in software programming, and all had >5 years of experience.

The overall CVI of the App was 0.99. The CVI score for most items was 1.00, except for "screen display" (0.98), "interaction" (0.98), "motivation" (0.98), and "documentation/help" (0.85). All CVI values exceeded the cutoff of 0.78. The expert judges analyzed the suggestions and comments from the prototype judges regarding modifications to the layouts and usability of the prototype.

After the modifications, the prototype was validated by 23 patients. Most participants were female (94.7%), with a median age of 52.1 years, an average weight of 84.3 kg (range, 65.7–110.4 kg), and an average BMI of 32.5 (24.2–45.4). Five (21.7%) were preoperative and 18 (78.3%) were postoperative bariatric surgery patients, all of whom underwent gastric bypass. Among postoperative patients, 15 (83.3%) were ≥12 months post-surgery, 2 (11.1%) were 6–12 months post-surgery, and 1 (5.5%) was <6 months post-surgery.

Target audience validation occurred over three rounds, with an overall mean agreement rate of 89.1%. There were good agreements in the following domains: layout, language, content organization, and engagement (Table 3).

**Table 3:** Validation of the prototype by the target audience

Questions	Answers			Concordance %
	"Yes"	"Partially"	"No"	
	n (%)	n (%)	n (%)	
1. Layout	21 (91.3)	2 (8.7)	0 (0)	91.3
1.1. Do you think the App's colors, images, texts, and letters suit its purpose?				
2. Language	20 (87.0)	3 (13.0)	0 (0)	87.0
2.1. Is the App easy to use?				
3. Content Organization	21 (91.3)	2 (8.7)	0 (0)	91.3
3.1 Is the amount of information on each screen appropriate to the target audience?				
4. Engagement	18 (78.2)	5 (21.8)	0 (0)	78.3
4.1 Do you think this application can increase user interest in this subject?				
4.2 Do you think you learned some new content using this application?	20 (87.0)	2 (8.7)	1 (4.3)	87.0
4.3 Would you recommend the application to other people?	23 (100.0)	0 (0)	0 (0)	100.0

Cultural adaptation also occurred over three rounds. In the first round, all suggestions were accepted and implemented by the expert committee. In the second round, the expert committee also agreed with the suggestions, but they were not implemented. For example, the target audience suggested excluding certain items such as dental prostheses from the preoperative hospital admission checklist outlined in the Learning Content subsection. However, the committee determined that, while specific needs may vary, the application aimed to provide comprehensive guidance for all patients. Therefore, the inclusion of all items on the preoperative admission checklist is crucial. Similarly, the target audience suggested that food should be delivered in smaller, more manageable portions. However, the committee opted not to incorporate this suggestion because of variations in hospital services and staffing limitations. Although participants also recommended adding specific physical activity plans, the committee retained the content to ensure applicability to all patients. Furthermore, the CEMSCBH does not have a physical activity professional for personalized workout prescriptions.

### Final version of the application

**Figure 2:**
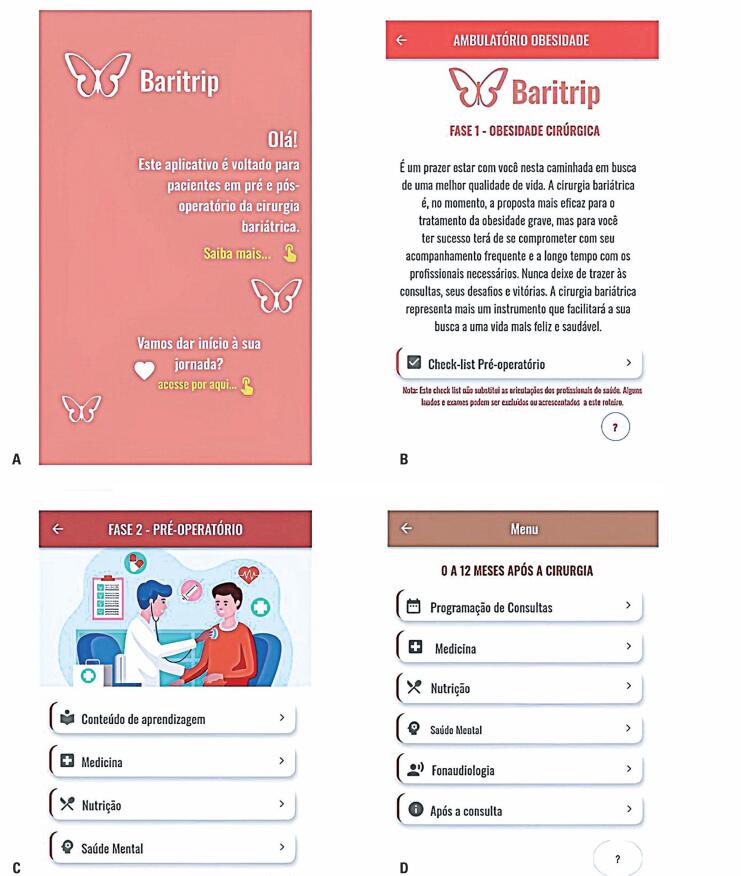
Screens of the Baritrip Mobile Application. (A) Welcome, (B) Introduction, (C) Preoperative, and (D) Postoperative (0–12 months) sections

The prototype began with the "Welcome" section that informs the users of the purpose and use of the App. After logging in, users start their journey on the "Home" screen, which provides access to the other sections and includes a "Help" button. This section comprises of 11 screens (Figure 2).

The "Introduction" section included four screens and introduced users to the CEM-SCBH outpatient service, including the required appointments and exams.

The "Preoperative" section included 19 screens covering content related to medicine, nutrition, and mental health. The content included preoperative information and education, indications for bariatric surgery, discharge instructions, and orientations to prevent alcohol and tobacco use. The Medicine subsection introduced the patient to the surgical process, explained what to expect, described various bariatric surgery techniques, and provided a list of items for hospital admission. It also provided postoperative care information. The Nutrition subsection introduced early postoperative nutritional care and provided a comprehensive nutrition plan as part of the assessment and preparation for bariatric surgery. The Mental Health subsection provided a comprehensive psychosocial evaluation, encompassing environmental, familial, nutritional, and behavioral factors. These evaluations complemented the information from in-person assessments. The Mental Health subsection included shared content with the "For your entire journey" section.

The "Postoperative: 0 to 12 months" section included 27 screens, starting with the Menu screen and ending with the "Congratulations" screen. It included subsections on Appointment Scheduling, Medicine, Nutrition, Mental Health, Speech Therapy, and After the Appointment. The medicine subsection described the complications of bariatric surgery and provided tips for reintroducing workouts. The nutritional subsection described the necessity for dietary reintroduction and texture progression during the postoperative period. The content promoted appropriate macro- and micronutrient intake, maximizing weight loss, and preserving lean mass. The Mental Health subsection provided guidance on quality of life, self-understanding, and active participation in treatment to build the body image of patients to give them security, improve their self-esteem, and alleviate signs of anxiety and depression.

The "Postoperative 12 to 24 months" section included 23 screens. It followed the same subsection structure as the prior section. The Nutrition subsection presented the Plate Method after bariatric surgery, which prioritized lean proteins, followed by vegetables and carbohydrates. The Medicine subsection described the causes and symptoms of dumping syndrome.

The "Beyond 24 months" section included 21 screens. It followed the same subsection structure as the prior section. The Nutrition subsection introduced a food diary to track the daily intake and habits of patients. The food diary helps understand the eating habits of patients undergoing bariatric surgery. This section also presented the types and indications for reconstructive plastic surgery. The Medicine subsection presented information on preventing weight regain and reminders to perform physical activities.

The "For your entire journey" section included 49 screens, including a quality-of-life questionnaire, calming techniques, sleep hygiene tips, weight loss goals, protein consumption monitoring tips, consultation preparation tips, and self-motivation strategies. It begins with a menu screen and concludes at the last screen of the self-motivation item.

The "Supplementary material" section included four screens, starting at the end of the "Beyond 24 months" section. It provided administrative information regarding medical appointments, motivational phrases, weight regain prevention tips, a website link to the Brazilian Association for the Study of Obesity and Metabolic Syndrome, and a playlist of relaxing music.

Gamification elements that were utilized in the App included reward messages, such as "You have completed the phase," "Congratulations," and visual elements like trophies and fireworks. After completing each section, users were directed to a "Congratulations, you have successfully completed" screen.

## DISCUSSION

mHealth Apps are revolutionizing healthcare services and promoting wellness by providing accessible health education and management tools. The logo and App name "Baritrip" symbolizes the bariatric journey and its transformation towards a healthy life.

Baritrip addresses the challenge of adapting scientific content to bariatric surgery. This includes aligning the content with the format and functionality constraints of mobile devices, such as screen size and processing performance. The App uses accessible language to achieve its goals of supporting both patients and healthcare professionals, effectively assisting users during the preoperative and postoperative phases of bariatric surgery.

As an emerging digital health intervention, the construction and viability of the Baritrip prototype application were part of a mature lifecycle. This study followed the mHealth Evidence Reporting and Assessment checklist to ensure standardized quality in digital health interventions.^(29)^ Although Baritrip is still in the initial stages of the maturity lifecycle (stage 2 of 6), it demonstrates high functionality fidelity and performance.^(30)^

Baritrip achieved a high CVI score of 0.99 overall. Previous studies suggest that a CVI ≥0.78 indicates adequacy for both individual items and overall application assessment.^(31)^ Comparable studies are few; however, a previous study used a questionnaire to evaluate nutritional supplementation, resulting in a CVI of 0.93. Using heuristics by De Carlo and Barbosa, we systematically evaluated various aspects of the App (design, content, and functionality), validating its suitability for educational purposes.^(25,32)^

The lower CVI scores for the Documentation and Help topics are possibly explained by the current developmental stage of the App. It is important to note that achieving optimal user performance also depends on hardware characteristics and internet speed. The prototype is still in the development stage, where it stores information and performs certain functions, but lacks full database integration; thus, the App uses simplified data handling and does not cover all possible error scenarios.

Cultural adaptation was ensured through faceto-face user interactions, aligning the App content with the cultural preferences and expectations of diverse user groups. This study followed the literature-recommended cultural adaptation steps; however, more standardized methods for conducting in-person testing are needed.^(33)^ The validation of the app by the target audience resulted in an adequate overall agreement score, comparable to previous validity studies.^(34,35)^

The benchmarking process revealed that most mHealth apps for patients undergoing bariatric surgery were in English. The following mHealth apps were available in Portuguese: Plate, BariFU, and Barilife. Although some apps may help bariatric surgery patients lose weight and improve other health outcomes, there is an opportunity to develop mHealth interventions to support preoperative and postoperative bariatric surgery patients in changing their behavior and improving outcomes. Baritrip, a novel multidisciplinary App, targets patients receiving public health care at SUS and offers a broader scope of educational resources and support than Apps such as Plate, BariUFU, and Barilife, which are more specialized in nutrition tracking and bariatric surgery support.

The uniqueness of Baritrip stems from the absence of prior instruments designed for bariatric surgery care and health education for SUS patients. It engages users throughout their preoperative and postoperative phases to monitor progress, enhance knowledge, improve self-care, track weight, and monitor quality of life, sleep habits, exercise routines, and anxiety levels. Gamification elements have been integrated into motivational strategies to foster understanding and knowledge acquisition throughout the different phases of bariatric surgery. The Baritrip App can potentially redefine learning for individuals undergoing bariatric surgery by integrating technology into their education. Designed for individuals with limited access to information, Baritrip emphasizes inclusion and cultural relevance. It represents a significant step in integrating technology into bariatric surgery education, preparing patients for long-term lifestyle changes.

The limitations of the prototype include the absence of functions to support personalized feedback and broader communication functionalities. Despite these limitations, the prototype remained functional and usable. Developing an App with relevant, practical, and reliable content while considering its acceptability and cost was challenging. Furthermore, managing schedules and navigating diverse professional opinions, especially amid pandemic-related challenges, presented more limitations.

## CONCLUSION

A multidisciplinary mHealth application prototype was developed and validated to enhance health literacy and promote self-care in patients undergoing bariatric surgery. The App uses simple and accessible language to support patient education and management.

## References

[1] Silvestrini B, Silvestrini M (2024). Physiopathology and Treatment of Obesity and Overweight: A Proposal for a New Anorectic. J Obes.

[2] The Lancet Gastroenterology Hepatology (2021). Obesity: another ongoing pandemic [editorial]. Lancet Gastroenterol Hepatol.

[3] Federation WO (2023). World Obesity Atlas 2023.

[4] World Health Organization (WHO) (2020). Overweight and obesity.

[5] Velapati SR, Shah M, Kuchkuntla AR, Abu-Dayyeh B, Grothe K, Hurt RT (2018). Weight Regain After Bariatric Surgery: Prevalence, Etiology, and Treatment. Curr Nutr Rep.

[6] Anekwe CV, Knight MG, Seetharaman S, Dutton WP, Chhabria SM, Stanford FC (2021). Pharmacotherapeutic options for weight regain after bariatric surgery. Curr Treat Options Gastroenterol.

[7] Bastos EC, Barbosa EM, Soriano GM, dos Santos EA, Vasconcelos SM (2013). Fatores determinantes do reganho ponderal no pós-operatório de cirurgia bariátrica. Arq Bras Cir Dig.

[8] Marcelino LF, Patrício ZM (2011). A complexidade da obesidade e o processo de viver após a cirurgia bariátrica: uma questão de saúde coletiva. Cien Saude Colet.

[9] Alexandrino EG, Marçal DF, Antunes MD, Oliveira LP, Massuda EM, Bertolini SM (2019). Physical activity level and lifestyle perception in prebariatric surgery patients. einstein (São Paulo).

[10] Monpellier VM, Janssen IM, Antoniou EE, Jansen AT (2019). Weight Change After Roux-en Y Gastric Bypass, Physical Activity and Eating Style: Is There a Relationship?. Obes Surg.

[11] Sørensen K, Van den Broucke S, Fullam J, Doyle G, Pelikan J, Slonska Z, Brand H, (HLS-EU) Consortium Health Literacy Project European (2012). Health literacy and public health: a systematic review and integration of definitions and models. BMC Public Health.

[12] Sousa CS, Turrini RN (2019). Desenvolvimento de aplicativo de celular educativo para pacientes submetidos à cirurgia ortognática. Rev Lat Am Enfermagem.

[13] Sørensen K (2024). Fostering digital health literacy to enhance trust and improve health outcomes. Comput Methods Programs Biomed Update.

[14] Nappo SA, Bigal AL (2021). Assessment of the level of health literacy of drug users in the Primary Health Care of the Brazilian Unified Health System. Res Soc Dev.

[15] Mangieri CW, Johnson RJ, Sweeney LB, Choi YU, Wood JC (2019). Mobile health applications enhance weight loss efficacy following bariatric surgery. Obes Res Clin Pract.

[16] Cheng J, Gao J, Shuai X, Wang G, Tao K (2016). The comprehensive summary of surgical versus non-surgical treatment for obesity: a systematic review and meta-analysis of randomized controlled trials. Oncotarget.

[17] Echer IC (2005). Elaboração de manuais de orientação para o cuidado em saúde. Rev Lat Am Enfermagem.

[18] Marjuni A, Azman MN, Mustofa HA, Sukadari S (2022). Development of the Android-Based Mobile Application "Mywheel Alignment" for Wheel Alignment Topics in Automotive Technology Courses at Vocational Colleges. AJVAH.

[19] Muda TZ, Wong XY, Teh XL (2022). Designing a domestic mobile apps: SAVIOR. JISTEM.

[20] Voglino C, Badalucco S, Tirone A, Ciuoli C, Cantara S, Benenati N (2022). Follow-up after bariatric surgery: is it time to tailor it? Analysis of early predictive factors of 3-year weight loss predictors of unsuccess in bariatric patients. Updates Surg.

[21] Alexandre NM, Coluci MZ (2011). Validade de conteúdo nos processos de construção e adaptação de instrumentos de medidas. Cien Saude Colet.

[22] Alves LF, Maia MM, Araújo MF, Damasceno MM, Freitas RW (2021). Desenvolvimento e validação de uma tecnologia MHEALTH para a promoção do autocuidado de adolescentes com diabetes. Cien Saude Colet.

[23] Marques AD, Moreira TM, Carvalho RE, Chaves EM, Oliveira SK, Felipe GF (2021). PEDCARE: validation of a mobile application on diabetic foot self-care. Rev Bras Enferm.

[24] Jasper MA (1994). Expert: a discussion of the implications of the concept as used in nursing. J Adv Nurs.

[25] D’Carlo D, Rodrigues Barbosa GA, de Oliveira ÉR (2017). Proposta de um Conjunto de Heurísticas para Avaliação da Usabilidade de Aplicativos Móveis Educacionais. Abakós.

[26] Cecilio SG (2016). Adequação cultural: Etapa complementar à tradução e adaptação de instrumentos em saúde [tese].

[27] Martins M, Campos C (2021). Protótipo de interface mobile para acompanhamento de pacientes de cirurgia bariátrica [tese].

[28] Hult M, Bonn SE, Andersson E, Spetz K, Lagerros YT (2018). The PromMera study – an RCT evaluating the effect of a smartphone application to improve lifestyle after bariatric surgery. Surg Obes Relat Dis.

[29] Agarwal S, LeFevre AE, Lee J, L’Engle K, Mehl G, Sinha C, Labrique A, WHO mHealth Technical Evidence Review Group (2016). Guidelines for reporting of health interventions using mobile phones: mobile health (mHealth) evidence reporting and assessment (mERA) checklist. BMJ.

[30] World Health Organization (WHO) (2016). Monitoring and evaluating digital health interventions: a practical guide to conducting research and assessment.

[31] Lopes LW, Alves GÂ, de Melo ML (2017). Content evidence of a spectrographic analysis protocol. Rev CEFAC.

[32] Abreu CA, Rosa JC, Matos ES (2020). Heurísticas de Usabilidade para Aplicativos Móveis Educacionais Infantis. Abakós.

[33] Neves SR, Dias Moreira A, Bandeira FF, Horta TG, Pagano AS, Reis JS (2023). Tradução, adaptação cultural e validação do Instrumento de Autoavaliação em Diabetes. REME.

[34] Bussmann JB, Martens WL, Tulen JH, Schasfoort FC, van den Berg-Emons HJ, Stam HJ (2001). Measuring daily behavior using ambulatory accelerometry: the Activity Monitor. Behav Res Methods Instrum Comput.

[35] Rodrigues LN, Santos AS, Gomes PP, Silva WC, Chaves EM (2020). Construction and validation of an educational booklet on care for children with gastrostomy. Rev Bras Enferm.

